# *CRY2* Genetic Variants Associate with Dysthymia

**DOI:** 10.1371/journal.pone.0071450

**Published:** 2013-08-08

**Authors:** Leena Kovanen, Mari Kaunisto, Kati Donner, Sirkku T. Saarikoski, Timo Partonen

**Affiliations:** 1 National Institute for Health and Welfare (THL), Department of Mental Health and Substance Abuse Services, Helsinki, Finland; 2 Institute for Molecular Medicine Finland (FIMM), University of Helsinki, Helsinki, Finland; 3 Folkhälsan Institute of Genetics, Folkhälsan Research Center, Helsinki, Finland; 4 Institute of Biomedicine, University of Helsinki, Helsinki, Finland; 5 Ministry of Social Affairs and Health, Department of Occupational Safety and Health, Helsinki, Finland; University of Wuerzburg, Germany

## Abstract

People with mood disorders often have disruptions in their circadian rhythms. Recent molecular genetics has linked circadian clock genes to mood disorders. Our objective was to study two core circadian clock genes, *CRY1* and *CRY2* as well as *TTC1* that interacts with *CRY2*, in relation to depressive and anxiety disorders. Of these three genes, 48 single-nucleotide polymorphisms (SNPs) whose selection was based on the linkage disequilibrium and potential functionality were genotyped in 5910 individuals from a nationwide population-based sample. The diagnoses of major depressive disorder, dysthymia and anxiety disorders were assessed with a structured interview (M-CIDI). In addition, the participants filled in self-report questionnaires on depressive and anxiety symptoms. Logistic and linear regression models were used to analyze the associations of the SNPs with the phenotypes. Four *CRY2* genetic variants (rs10838524, rs7121611, rs7945565, rs1401419) associated significantly with dysthymia (false discovery rate q<0.05). This finding together with earlier *CRY2* associations with winter depression and with bipolar type 1 disorder supports the view that *CRY2* gene has a role in mood disorders.

## Introduction

Circadian rhythms are about 24-hour oscillations in physiological processes that allow humans to anticipate routine changes in the environment. The rhythms are generated by the internal clock, the principal one being located in the suprachiasmatic nucleus of the anterior hypothalamus in the brain. At the molecular level, the transcription of a network of genes is switched on and off following the approximate 24-hour pattern. A feedback loop is initiated by binding of CLOCK protein [1] with aryl hydrocarbon receptor nuclear translocator-like (ARNTL) protein [2]. The heterodimer then activates the transcription of a large set of genes and among them the period (*PER1*, *PER2*, *PER3*) and cryptochrome (*CRY1*, *CRY2*) genes. Again, the encoded proteins form heterodimers (PER-with-CRY dimers) which translocate back to the nucleus and inhibit the CLOCK-with-ARNTL mediated transcription, thereby repressing their own transcription [3]. In addition, nuclear receptors together with their co-activators and co-repressors feed back to ARNTL [4].

The cryptochrome genes may be of key importance here, because of their unique role in the core of these feedback loops [3], [5], [6]. The nuclear ratio of CRY1 to CRY2 proteins controls the period of the circadian clock [5] and the effects of CRY2 on circadian regulation are sensitive to the dosage of CRY1 [7]. Findings from knock-out mice suggest that without *CRY2* the circadian period is lengthened similar to “evening owls”, whereas without *CRY1* it is shortened [8], [9]. Furthermore, CRY2 appears to play a critical role in tuning the suprachiasmatic nucleus to 24 hour cycle by opposing and titrating the decelerating action of CRY1 [7].

“Evening owls” are known to have increased risk for anxiety and depressive symptoms [10], [11] that may develop into depressive disorders [12], [13]. Furthermore, dysfunction of signaling pathways involving CRY1 and CRY2 might lead into depressive behaviors as follows. Interaction of CRY1 and CRY2 proteins directly with the Gsα subunit of heterotrimeric G protein [14] and with adenylyl cyclase [15] reduces the accumulation of cyclic adenosine monophosphate in response to G protein-coupled receptor activation and inhibits downstream reactions [15], [16]. If this inhibition is not appropriate, the inhibitory tone on cyclic adenosine monophosphate signaling is overruled, leading to the activation of cascades downstream, such as cyclic adenosine monophosphate response element-binding protein activity, and to the increase in depressive-like behaviors [17].

Disrupted or misaligned circadian rhythms and circadian gene expression are often found in major depressive disorder and bipolar disorder, with or without a seasonal pattern [18], [19]. Since the original finding [20], there is a growing evidence that genetic variations in the core circadian clock genes associate with mood and anxiety disorders [21]. In addition, these genetic variations associate with sleep and metabolic disorders [22] that are often comorbid conditions with mood and anxiety disorders. With the focus on the cryptochrome genes, earlier studies have indicated *CRY1* genetic variants in major depressive disorder [23] and *CRY2* genetic variants in seasonal affective disorder, winter depression in particular, and in bipolar type 1 disorder [24], [25].

The aim of the current paper is to study the associations of *CRY1*, *CRY2* and *TTC1* genes in depressive and anxiety disorders. In addition to the two cryptochrome genes, *TTC1* was selected in order to analyze epistasis, if any, between the *TTC1* and *CRY2* genes, because the TTC1 protein interacts physically with CRY2 [26]. The present work extends the earlier studies by deeper linkage disequilibrium (LD) coverage of the genes, by including potential functional variants, by using a large sample derived randomly from the general population, by assessment based on both a diagnostic interview and self-reports, and by analysis of diverse phenotype information. Here we report a significant association of *CRY2* genetic variants with dysthymia.

## Materials and Methods

### Ethics Statement

The ethics committees of the National Public Health Institute and the Helsinki and Uusimaa Hospital District accepted the study protocol and its ethical approval, and all participants provided a written informed consent.

### Subjects

This study was part of a nationwide health interview and examination survey, Health 2000. The survey included a health status examination and the Munich-Composite International Diagnostic Interview (M-CIDI) [27]. M-CIDI is a valid and reliable instrument for the assessment of depressive, anxiety and alcohol use disorders, yielding diagnoses according to Diagnostic and Statistical Manual of Mental Disorders, Fourth Edition (DSM-IV). In addition, participants gave venous blood samples for DNA extraction and filled in two sets of questionnaires. The sample for our current study from the Health 2000 participants aged 30 years and older (n = 8028) included 5910 individuals (3283 women, 2627 men) who had given blood samples, taken part to the M-CIDI interview and filled in the self-report on seasonal changes in mood and behavior. The study design and methods have been described in detail elsewhere (http://www.terveys2000.fi/indexe.html).

### Phenotypes

Altogether 12 phenotypes related to mental well-being (diagnoses according to DSM-IV and psychometric scales) were analyzed (Table 1). Mental disorders analyzed in the current study included major depressive disorder, dysthymia, depressive disorders (major depressive disorder, dysthymia), panic disorder, anxiety disorders (panic disorder w/o agoraphobia, generalized anxiety disorder, social phobia, agoraphobia), and the comorbid conditions of depressive, anxiety and alcohol use disorders (alcohol dependence and abuse), as assessed with the M-CIDI. The controls did not have any diagnosis of mental disorders nor met any sub-threshold criteria as assessed with the M-CIDI.

**Table 1:** Number of subjects in mental health related phenotypes (DSM-IV based diagnoses and quantitative scores on psychometric scales) analyzed in the study.

	All		Women		Men	
Phenotypes analyzed	Cases	Controls	Cases	Controls	Cases	Controls
Depressive disorders	354	3871	242	2135	112	1736
Major depressive disorder	267	3871	183	2135	84	1736
Dysthymia	136	3871	90	2135	46	1736
Anxiety disorders	221	3871	137	2135	84	1736
Panic disorder	106	3871	71	2135	35	1736
Comorbid depressive disorders	145	3871	84	2135	61	1736
Comorbid anxiety disorders	117	3871	63	2135	54	1736
Comorbid alcohol use disorders	105	3871	38	2135	67	1736
BDI	5724		3153		2571	
GHQ	5811		3211		2600	
MBI	3379		1745		1634	
GSS	5633		3096		2537	

BDI; Beck Depression Inventory.

GHQ; General Health Questionnaire.

MBI; Maslach Burnout Inventory.

GSS; Global Seasonality Score.

In addition to attending the diagnostic interview, the participants completed the following four self-reports that were also analyzed: 1) a modified 21-item Beck Depression Inventory (BDI; [28]), as adapted and validated for the Finnish population [29], 2) the 16-item Maslach Burnout Inventory (MBI; [30]); 3) the 12-item General Health Questionnaire (GHQ; [31]), and 4) a modified [32] 7-item Seasonal Pattern Assessment Questionnaire (SPAQ; [33]). These questionnaires give quantitative information about depressive and anxiety symptoms.

### Gene and SNP Selection

Three genes of interest were selected: *CRY1*, *CRY2* and *TTC1*. The SNP selection was based on phase 3 data of the HapMap database (http://www.hapmap.org/) and done using the Tagger program included in the Haploview 4.1 software [34]. Information about the LD within the chosen areas of the genome was used to select an optimal set of SNPs capturing most of the genetic variation. Areas of the genome covered were the genes and 10 kb of their 5′ and 3′ flanking regions. For *CRY1*, this area of interest was 122 kb (chr12∶105,899–106,021 kb, NCBI36/hg18 assembly) in size, whereas for *CRY2* it was 56 kb (chr11∶45,815–45,871 kb) and for *TTC1* 77 kb (chr5∶159,358–159,435 kb).

The HapMap database included 21, 34 and 15 SNPs having a minor allele frequency (MAF) >5% in the European population (CEU and TSI) for the areas containing the *CRY1*, *CRY2* and *TTC1* genes, respectively. The aim was to capture all these SNPs by setting the limit for the pair-wise r^2^ to ≥0.9. Based on this criterion, ten *CRY1* SNPs, ten *CRY2* SNPs and nine *TTC1* SNPs were decided to be genotyped and all, except one (*TTC1* rs3733868), were successfully included in the genotyping multiplexes. One SNP (*CRY1* rs11113153) failed in the genotyping phase.

In addition to the aforementioned SNPs, potentially functional variants, as many as possible, were included in the genotyping multiplexes. These 21 additional SNPs (8 for *CRY2*, 11 for *CRY1* and 2 for *TTC1*) were selected using Pupasuite [35], Variowatch [36], dbSMR [37] and miRNASNP [38] databases. Table 2 presents all the 48 SNPs that were successfully genotyped in this study and their selection criteria.

**Table 2:** Successfully genotyped SNPs, their selection criteria, allele and genotype frequencies, and Hardy-Weinberg equilibrium p-values.

Gene	SNP	BP	A_1_	A_2_	MAF	A_1_A_1_	A_1_A_2_	A_2_A_2_	HWE-P	Selection criteria
*TTC1*	rs6878309	159358102	*C*	*T*	0.09	57 (0.01)	990 (0.17)	4800 (0.82)	0.44	LD
*TTC1*	rs1402024	159361425	*G*	*A*	0.02	1 (0)	177 (0.03)	5636 (0.97)	1	LD
*TTC1*	rs41275305	159370449	*G*	*C*	0	0 (0)	31 (0.01)	5815 (0.99)	1	non-synonymous coding variant
*TTC1*	rs10515804	159380041	*T*	*C*	0.3	531 (0.09)	2468 (0.43)	2797 (0.48)	0.71	LD
*TTC1*	rs2176830	159380714	*A*	*G*	0.07	32 (0.01)	756 (0.13)	5057 (0.87)	0.48	LD
*TTC1*	rs12520927	159410735	*G*		0	0 (0)	0 (0)	5854 (1)	1	non-synonymous coding variant
*TTC1*	rs3733869	159422128	*T*	*C*	0.3	522 (0.09)	2430 (0.42)	2866 (0.49)	0.83	LD
*TTC1*	rs6861719	159422825	*T*	*C*	0.23	295 (0.05)	2066 (0.36)	3448 (0.59)	0.55	LD
*TTC1*	rs1106055	159424098	*A*	*G*	0.32	607 (0.1)	2517 (0.43)	2674 (0.46)	0.7	LD
*TTC1*	rs7715826	159425636	*T*	*C*	0.02	5 (0)	254 (0.04)	5595 (0.96)	0.22	LD
*CRY2*	rs7121611	45820718	*A*	*T*	0.46	1218 (0.21)	2883 (0.5)	1711 (0.29)	0.96	LD
*CRY2*	rs7121775	45820899	*C*	*T*	0.27	384 (0.07)	2326 (0.4)	3100 (0.53)	0.06	LD
*CRY2*	rs61884508	45821508	*G*	*T*	0.02	1 (0)	241 (0.04)	5600 (0.96)	0.52	Pupasuite OregannoFilter TFBS
*CRY2*	rs75065406	45821518	*T*	*C*	0.04	13 (0)	421 (0.07)	5414 (0.93)	0.11	TFBS, MAF
*CRY2*	rs3747548	45825589	*A*	*C*	0	0 (0)	1 (0)	5847 (1)	1	Pupasuite non-synonymous & VarioWatch
*CRY2*	rs10838524	45826753	*G*	*A*	0.48	1337 (0.23)	2897 (0.5)	1579 (0.27)	0.92	LD & Lavebratt et al.
*CRY2*	rs2292913	45834105	*T*	*C*	0.05	18 (0)	590 (0.1)	5233 (0.9)	0.7	LD & splice site
*CRY2*	rs7945565	45835568	*G*	*A*	0.46	1213 (0.21)	2890 (0.5)	1695 (0.29)	0.79	Pupasuite Triplex
*CRY2*	rs1401419	45836315	*G*	*A*	0.46	1211 (0.21)	2909 (0.5)	1681 (0.29)	0.48	Pupasuite Triplex
*CRY2*	rs72902437	45838834	*C*	*T*	0.03	2 (0)	313 (0.05)	5499 (0.95)	0.45	Pupasuite Triplex
*CRY2*	rs35488012	45845804	*G*		0	0 (0)	0 (0)	5854 (1)	1	Variowatch synonymous
*CRY2*	rs7123390	45847994	*A*	*G*	0.29	431 (0.07)	2445 (0.42)	2915 (0.5)	0.01	LD & Lavebratt et al.
*CRY2*	rs4755345	45848084	*A*	*G*	0.05	18 (0)	598 (0.1)	5229 (0.89)	0.8	LD
*CRY2*	rs17787136	45851212	*G*	*C*	0.28	409 (0.07)	2385 (0.41)	3014 (0.52)	0.03	Pupasuite TFBS
*CRY2*	rs10838527	45859770	*G*	*A*	0.12	89 (0.02)	1236 (0.21)	4509 (0.77)	0.67	LD & Lavebratt et al.
*CRY2*	rs2292910	45860189	*A*	*C*	0.34	650 (0.11)	2707 (0.47)	2455 (0.42)	0.02	LD & dbSMR miRNA target site
*CRY2*	rs3824872	45862181	*T*	*G*	0.25	372 (0.06)	2173 (0.37)	3276 (0.56)	0.65	LD & Lavebratt et al.
*CRY2*	rs1554338	45863406	*G*	*A*	0.05	14 (0)	528 (0.09)	5289 (0.91)	0.77	LD
*CRY1*	rs4964513	105899888	*C*	*T*	0.12	84 (0.01)	1224 (0.21)	4492 (0.77)	0.95	LD
*CRY1*	rs714359	105902975	*A*	*G*	0.22	276 (0.05)	2006 (0.35)	3516 (0.61)	0.67	LD
*CRY1*	rs12821586	105904582	*A*	*G*	0.11	72 (0.01)	1138 (0.19)	4629 (0.79)	0.84	LD
*CRY1*	rs2287161	105905270	*C*	*G*	0.5	1408 (0.24)	2930 (0.51)	1461 (0.25)	0.43	Soria et al. & Utge et al. & Pupasuite triplex
*CRY1*	rs8192441	105909584	*C*	*A*	0.01	1 (0)	136 (0.02)	5712 (0.98)	0.56	miRNASNP miRNA target site
*CRY1*	rs3741892	105911293	*C*	*G*	0.49	1395 (0.24)	2937 (0.51)	1477 (0.25)	0.4	Pupasuite Triplex
*CRY1*	rs10861688	105918178	*T*	*C*	0.17	164 (0.03)	1642 (0.28)	4011 (0.69)	0.82	LD
*CRY1*	rs10861697	105943792	*C*	*G*	0.49	1352 (0.23)	2923 (0.5)	1521 (0.26)	0.48	Pupasuite Triplex
*CRY1*	rs2078074	105960936	*C*	*T*	0.42	1017 (0.18)	2815 (0.49)	1930 (0.33)	0.87	Pupasuite Transfac
*CRY1*	rs59790130	105964433	*T*	*C*	0.06	26 (0)	692 (0.12)	5131 (0.88)	0.58	Pupasuite Transfac
*CRY1*	rs10437895	105964954	*C*	*T*	0.49	1398 (0.24)	2936 (0.5)	1482 (0.25)	0.46	Pupasuite Transfac
*CRY1*	rs10746077	105965682	*A*	*G*	0.42	1027 (0.18)	2832 (0.49)	1960 (0.34)	0.96	Pupasuite Transfac
*CRY1*	rs11613557	105966445	*T*	*C*	0.06	26 (0)	692 (0.12)	5130 (0.88)	0.58	Pupasuite Triplex
*CRY1*	rs2888896	105970712	*T*	*C*	0.42	1019 (0.18)	2830 (0.49)	1947 (0.34)	0.87	LD
*CRY1*	rs11113179	105976915	*T*	*C*	0.08	39 (0.01)	833 (0.14)	4942 (0.85)	0.53	LD & Utge et al.
*CRY1*	rs10746083	105978532	*T*	*C*	0.49	1391 (0.24)	2941 (0.51)	1481 (0.25)	0.37	Pupasuite Triplex
*CRY1*	rs4964518	105990347	*T*	*C*	0.07	30 (0.01)	778 (0.13)	5027 (0.86)	1	LD
*CRY1*	rs7294758	105991959	*A*	*T*	0.01	0 (0)	97 (0.02)	5758 (0.98)	1	Pupasuite Triplex
*CRY1*	rs17289712	105993098	*G*	*A*	0.05	6 (0)	524 (0.09)	5308 (0.91)	0.07	LD
*CRY1*	rs10778528	105998092	*G*	*T*	0.48	1358 (0.23)	2926 (0.5)	1533 (0.26)	0.62	LD

BP; Base pair position based on NCBI36/hg18 build.

A_1_; Minor allele.

A_2_; Major allele.

MAF; Minor allele frequency.

A_1_A_1_, A_1_A_2_, A_2_A_2_; genotype counts and frequencies (%).

HWE-P; Hardy-Weinberg equilibrium p-value.

LD; Linkage disequilibrium.

TFBS, Transcription factor binding site.

### Genotyping

Genomic DNA was isolated from the whole blood according to standard procedures. DNA samples were available from 5910 subjects for this study. The SNPs were genotyped using the Sequenom MassARRAY system and the iPLEX Gold Single Base Extension chemistry (Sequenom, San Diego, CA, USA) in a multiplex format [39]. This method has excellent success (>95%) and accuracy (100%) rates [40]. For the genotyping quality control purposes, both positive (CEPH) and negative water controls were included in each 384-plate. Genotyping was performed blind to phenotypic information.

173 individuals were removed from the statistical analyses due to a high missing genotype rate (i.e. >0.1). The total genotyping rate in the remaining individuals was 0.9987. In addition, the following five SNPs were removed because of the minor allele frequency of <0.01: rs41275305, rs12520927, rs3747548, rs35488012, rs7294758. Finally, there were 5737 individuals and 43 SNPs for the statistical analyses.

### Statistical Analyses

Statistical analyses were performed with the PLINK software [41], release v1.07. The additive model was calculated using logistic and linear regression models controlling for age and sex. In addition, dominant and recessive models were calculated and the analyses were performed in both sexes separately. For continuous phenotypes 10,000 permutations were used to produce empirical p-values in order to relax the assumption of normality. The results were corrected for multiple testing across all the tests (SNPs, phenotypes, gender, genetic models) by calculating false discovery rate (FDR) q-values [42] using R software (http://www.r-project.org/). The q-values of <0.05 were considered significant.

Haploview software [34] was used to define haplotype blocks by the confidence interval method. Each haplotype in the formed blocks was analyzed by PLINK software using linear and logistic regression, additive model and controlling for age and sex. Sexes were also analyzed separately. Continuous phenotypes were permuted 10,000 times. Interactions between *CRY2* and *TTC1* (SNP × SNP epistasis) were tested and calculated using the PLINK software.

## Results

The genotype and allele frequencies and Hardy-Weinberg equilibrium (HWE) estimates are shown in Table 2 (see Table S1 for the genotype counts by phenotype). The results of the association analyses where q<0.15 are presented in Table 3 and the remaining results of the association analyses in Table S2. Four *CRY2* SNPs showed evidence of association with dysthymia using the additive model (rs10838524 risk allele *G*, p = 0.000020, OR = 1.75; rs7121611 risk allele *A*, p = 0.000022, OR = 1.74; rs7945565 risk allele *G*, p = 0.000039, OR = 1.71; rs1401419 risk allele *G*, p = 0.000041, OR = 1.71). *CRY2* SNP rs10838524 showed evidence of association also using the dominant model (p = 0.000059, OR = 3.27) and the additive model for women (p = 0.000026, OR = 1.97). The *CRY1* and *TTC1* genetic variants studied showed no evidence of association with any of the phenotypes analyzed. No epistasis between *CRY2* and *TTC1* SNPs was observed.

**Table 3:** Results from the single SNP association analyses (q<0.15).

Phenotype	Model	Gene	SNP	A1	Odds ratio	L95	U95	P-value	Q-value
Dysthymia	ADD	*CRY2*	rs10838524	*G*	1.75	1.35	2.27	2.00E-05	0.04*
Dysthymia	ADD women	*CRY2*	rs10838524	*G*	1.97	1.44	2.71	2.60E-05	0.04*
Dysthymia	DOM	*CRY2*	rs10838524	*G*	3.27	1.84	5.83	5.90E-05	0.04*
Dysthymia	DOM women	*CRY2*	rs10838524	*G*	5.11	2.22	11.77	0.00013	0.07
									
Dysthymia	ADD	*CRY2*	rs1401419	*G*	1.71	1.32	2.21	4.10E-05	0.04*
Dysthymia	ADD women	*CRY2*	rs1401419	*G*	1.82	1.33	2.48	0.00018	0.07
Dysthymia	DOM	*CRY2*	rs1401419	*G*	2.67	1.59	4.46	0.0002	0.07
Dysthymia	DOM women	*CRY2*	rs1401419	*G*	3.20	1.65	6.23	0.00061	0.14
Depressive disorders	DOM	*CRY2*	rs1401419	*G*	1.62	1.23	2.13	0.00063	0.14
									
Dysthymia	ADD	*CRY2*	rs3824872	*T*	0.52	0.37	0.74	0.00024	0.08
									
Dysthymia	ADD	*CRY2*	rs7121611	*A*	1.74	1.35	2.24	2.20E-05	0.04*
Dysthymia	ADD women	*CRY2*	rs7121611	*A*	1.86	1.36	2.53	9.40E-05	0.06
Dysthymia	DOM	*CRY2*	rs7121611	*A*	2.74	1.63	4.58	0.00013	0.07
Depressive disorders	DOM	*CRY2*	rs7121611	*A*	1.66	1.26	2.18	0.00033	0.1
Dysthymia	DOM women	*CRY2*	rs7121611	*A*	3.30	1.7	6.42	0.00043	0.12
									
Dysthymia	ADD	*CRY2*	rs7945565	*G*	1.71	1.33	2.21	3.90E-05	0.04*
Dysthymia	ADD women	*CRY2*	rs7945565	*G*	1.82	1.33	2.49	0.00017	0.07
Dysthymia	DOM	*CRY2*	rs7945565	*G*	2.68	1.6	4.49	0.00018	0.07
Depressive disorders	DOM	*CRY2*	rs7945565	*G*	1.65	1.25	2.17	0.00042	0.12
Dysthymia	DOM women	*CRY2*	rs7945565	*G*	3.22	1.65	6.26	0.00058	0.14

ADD; Additive model.

DOM; Dominant model.

A1; Tested allele (minor allele).

L95, U95; Lower and upper bounds of 95% confidence interval for odds ratio.

* Significant association.

**Figure 1:**
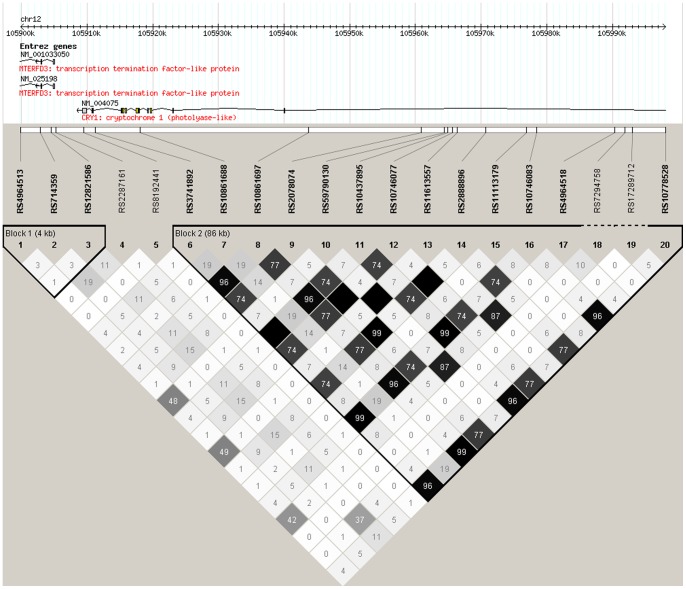
The analyzed *CRY1* SNPs in this study, their location and the haplotype block structure of the area formed based on our sample showing r^2^ values. The confidence interval algorithm implemented in the Haploview program was used to construct the haplotype blocks.

**Figure 2:**
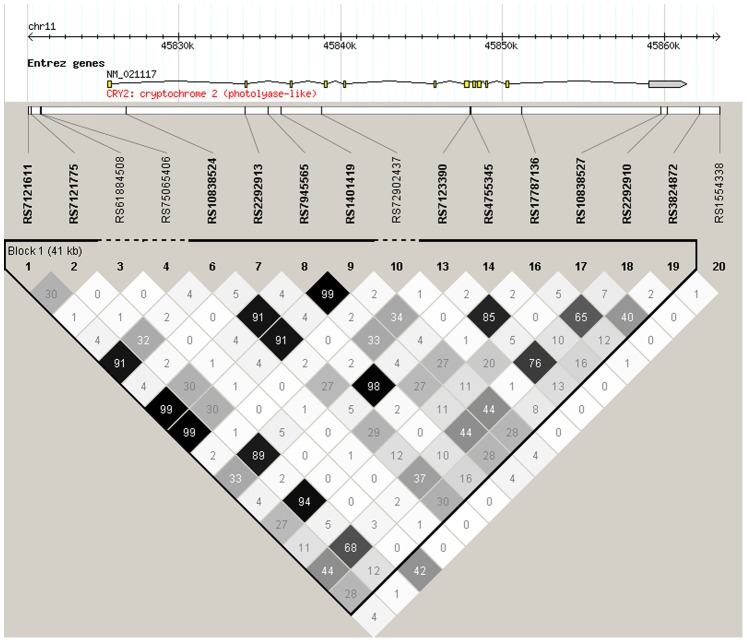
The analyzed *CRY2* SNPs, their location and the haplotype block structure constructed using the Haploview program showing r^2^ values.

**Figure 3:**
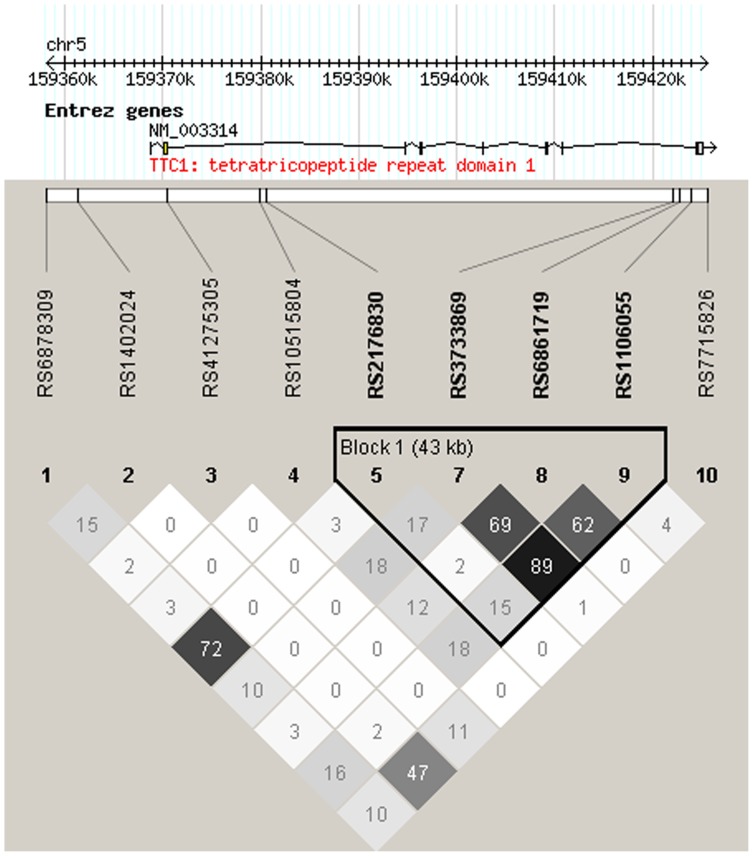
The analyzed *TTC1* SNPs, their location and the haplotype block structure constructed using the Haploview program showing r^2^ values.

Haploview produced two haplotype blocks for *CRY1* (Figure 1), one for *CRY2* (Figure 2) and one for *TTC1* (Figure 3). The haplotype analyses supported the association of *CRY2* with dysthymia, as the ATTCGCGGTGGCACG haplotype containing the risk alleles *A*, *G*, *G* and *G* of SNPs rs7121611, rs10838524, rs7945565 and rs1401419, respectively, showed association with dysthymia in all cases (p = 0.00034, OR = 1.58) and among women (p = 0.0011, OR = 1.66). Table 4 presents the haplotype associations with p-values (or empirical p-values) of <0.010.

**Table 4:** Results from the haplotype association analyses (p<0.10).

Phenotype	Population	NSNP	NHAP	Gene	SNP1	SNP2	Haplotype	Frequency	Odds ratio	p-value	Empirical p-value
Dysthymia	all	15	10	*CRY2*	rs7121611	rs3824872	*ATTCGCGGTGGCACG*	0.41	1.58	0.00034	
Dysthymia	women	15	10	*CRY2*	rs7121611	rs3824872	*ATTCGCGGTGGCACG*	0.407	1.66	0.0011	
Depressivedisorders	men	3	4	*CRY1*	rs4964513	rs12821586	*TAG*	0.217	1.67	0.0014	
Depressivedisorders	all	15	10	*CRY2*	rs7121611	rs3824872	*ATTCGCGGTGGCACG*	0.41	1.27	0.0039	
Depressivedisorders	women	15	10	*CRY2*	rs7121611	rs3824872	*TTTCATAATGACAAT*	0.0539	0.45	0.0057	
Depressivedisorders	all	15	10	*CRY2*	rs7121611	rs3824872	*TTTCATAATGACAAT*	0.0514	0.53	0.0061	
Dysthymia	all	15	10	*CRY2*	rs7121611	rs3824872	*TTTCATAATGACAAT*	0.0514	0.2	0.0065	
Major depressivedisorder	men	3	4	*CRY1*	rs4964513	rs12821586	*TAG*	0.217	1.62	0.0082	
GSS	all	14	6	*CRY1*	rs3741892	rs10778528	*GTGTCTGCCCCCAT*	0.17	0.2	0.011	0.01

NSNP; Number of SNPs in this haplotype.

NHAP; Number of common haplotypes (f>0.01).

SNP1; SNP ID of the first SNP (5′).

SNP2; SNP ID of the last SNP (3′).

## Discussion

Our main finding was that *CRY2* genetic variants associated with dysthymia in our random sample derived from the general population aged 30 years and older. The associations with dysthymia were stronger in the additive than in the dominant model and became weaker when we analyzed only women (n = 88). No association was observed in men. The sample size for dysthymia was limited (n = 128) and the lack of association in men could be due to a small sample size (n = 40). We did not find any significant association of *CRY2* with major depressive disorder, whether with a single or recurrent episode, or with anxiety disorders, and there was no significant association of *CRY1* or *TTC1* with any of the phenotypes assessed.

Of the individual *CRY2* SNPs, rs7121611 (upstream), rs10838524 (intron 1), rs7945565 (intron 2) and rs1401419 (intron 2) showed evidence of association with dysthymia, with the *A*, *G*, *G* and *G* alleles being predisposing, respectively. These associations are supported by the haplotype analysis, as the *CRY2* ATTCGCGGTGGCACG haplotype including the individual risk alleles associated with the phenotype. The associated haplotype of *CRY2* spans almost the whole gene and the four associated SNPs are located in the first half of the haplotype block (Figure 2). According to transcription factor CHiPSeq from ENCODE (http://genome.ucsc.edu/ENCODE/) SNP rs10838524 is located in a regulatory region and according to Pupasuite software [35] rs7945565 and rs1401419 were predicted to be putative triplex disrupting SNPs which may also be part of regulatory regions for controlling gene expression [43].

Of the four associated *CRY2* SNPs, only rs10838524 has previously been linked to mental health disorders. Concerning this SNP, our current results are partly in line with the findings of Lavebratt et al. [24] who found the same risk allele for winter depression in the Finnish subpopulation of their study. The allele frequencies in cases and controls were similar in these two sets, as expected for the controls, since they were drawn from the same Health 2000 cohort. The cases, on the other hand, had been recruited from outpatient services in the capital area [24]. Another Finnish study, using a population based sample also from the Health 2000 cohort, found the same minor allele to weakly associate with depressive disorders in women and in women having depressive disorders with early morning awakening, and the association was supported with haplotype analyses but did not hold after correcting for multiple testing [44]. We also analyzed depressive disorders in women and found similar crude p-value (p = 0.035, Table S2). In the study by Utge et al., the statistical analyses had been done separately in men and women. In our analyses, the associations were stronger when using the dominant model in both sexes together (p = 0.0010), but still the association to depressive disorders did not reach statistical significance after correcting for multiple testing. For the Swedish subpopulation of the winter depression study [24] as well as for a Swedish bipolar study [25], the allele frequencies for *CRY2* rs10838524 were reversed and the other allele, i.e. *A*, was found as the risk allele. Earlier, the *A* allele has also been associated with a greater degree of chronic course of depressive symptoms in a sample of 35 individuals with major depression or bipolar disorder [45].

Concerning SNP rs3824872, located in the 3′ region of *CRY2* (but also in the 5′ region of *MAPK8IP1* gene), our study suggests (q = 0.08) that the *T* allele is the protective one against dysthymia, and a similar (though non-significant) tendency is seen in the Finnish subpopulation of the winter depression study [24], while in the Swedish subpopulation of the same study, the other allele, i.e. *A*, was the risk allele. With regard to *CRY2* rs7123390, the SNP was earlier associated with winter depression in the Finnish subpopulation [24], but in the current study, we did not observe any significant association with this intronic SNP. Moreover, there was no significant association with the 3′UTR *CRY2* rs10838527, similar to earlier findings where no association with winter depression was observed among the Finnish subpopulation, whereas this SNP did associate with winter depression among the Swedish subpopulation [24].

Although preliminary, our analysis has demonstrated that *CRY2* associates robustly with dysthymia which is characterized by a chronic course of illness where a depressive episode lasts for two years or longer and often deepens into a major depressive episode. Earlier, *CRY2* genetic variants have been associated with winter recurrent depressive disorder [24], and with the rapid cycling of bipolar type 1 disorder where depressive episodes dominate the clinical course of illness [25]. A single *CRY1* variant has been associated with major depressive disorder earlier [23] but there is no replication of the finding [21]. Furthermore, it has been previously observed that following the antidepressant sleep deprivation, the expression of *CRY2* mRNA increased in controls, whereas no change or response to the treatment was observed in patients with bipolar disorder whose *CRY2* mRNA levels were markedly lower than in controls [24]. Moreover in an animal study, *CRY2* mRNA expression was abnormal when inbred-strain mice with the intrinsic level of high anxiety were deprived of sleep [46]. Currently, it is however not known, whether the knock-out or the knock-down of *CRY2* in the whole organism or specific to a tissue produces any change in anxiety-like or depressive-like behaviors, or how mutated CRY2 proteins influence mood-related behaviors. *CRY2* and *CRY1* proteins are functionally different [7] which could impact their circadian outputs differently and make a difference in the consequent phenotype. This difference could explain why *CRY2*, not *CRY1*, associated with dysthymia in our study. Moreover, it might be that *CRY2* variants play a leading role in circadian hierarchy [3] and thereby in the pathogenesis of mood disorders as well. Some circadian clock gene variants seem to have a role in neuropsychiatric disorders in general [47], [48]. Elucidating the mechanisms of action with these genetic variants might therefore benefit the development of treatments for and beyond mood and anxiety disorders [18], [49], [50].

A limitation in our current epidemiological health examination study is that the seasonal pattern, whether in major depressive disorder or bipolar disorder, was not assessed at all. Another limitation is that the diagnoses of bipolar disorders were considered not adequate for analysis. However, it is of note that bipolar type 1 disorder is markedly less frequent in Finland compared with the prevalence in many Western-type societies [51].

To conclude, *CRY2* genetic variants associated with dysthymia. This new data, together with the earlier findings, reinforces the involvement of *CRY2* in mood disorders.

## Supporting Information

Table S1 **Genotype counts and Hardy-Weinberg equilibrium p-values for successfully genotyped SNPs in the analyzed phenotypes.**
(XLS)

Table S2 **Results from the single SNP and haplotype association analyses.**
(XLS)
